# Integrative analysis of key candidate genes and signaling pathways in autoimmune thyroid dysfunction related to anti-CTLA-4 therapy by bioinformatics

**DOI:** 10.1007/s10637-020-00952-z

**Published:** 2020-06-04

**Authors:** Ying Zhang, Francesca Garofano, Xiaolong Wu, Matthias Schmid, Peter Krawitz, Markus Essler, Ingo G. H. Schmidt-Wolf

**Affiliations:** 1 Department of Integrated Oncology, Center for Integrated Oncology (CIO), University Hospital Bonn, Venusberg-Campus 1, D-53127 Bonn, Germany; 2 Institute for Medical Biometry, Computer Science and Epidemiology, University Hospital Bonn, Bonn, Germany; 3 Institute for Genomic Statistics and Bioinformatics of Medical School University Bonn, Bonn, Germany; 4 Department of Nuclear Medicine, University Hospital Bonn, Bonn, Germany

**Keywords:** Immune checkpoint blockade, CTLA-4, Autoimmune thyroid dysfunction, Differentially expressed genes, Signaling pathway

## Abstract

Cytotoxic T lymphocyte-associated antigen-4 (CTLA-4), the first immune checkpoint to be targeted clinically, has provided an effective treatment option for various malignancies. However, the clinical advantages associated with CTLA-4 inhibitors can be offset by the potentially severe immune-related adverse events (IRAEs), including autoimmune thyroid dysfunction. To investigate the candidate genes and signaling pathways involving in autoimmune thyroid dysfunction related to anti-CTLA-4 therapy, integrated differentially expressed genes (DEGs) were extracted from the intersection of genes from Gene Expression Omnibus (GEO) datasets and text mining. The functional enrichment was performed by gene ontology (GO) annotation and Kyoto encyclopedia of genes and genomes (KEGG) pathway analysis. Protein-protein interaction (PPI) network, module enrichment, and hub gene identification were constructed and visualized by the online Search Tool for the Retrieval of Interacting Genes (STRING) and Cytoscape software. A total of 22 and 17 integrated human DEGs in hypothyroidism and hyperthyroidism group related to anti-CTLA-4 therapy were identified, respectively. Functional enrichment analysis revealed 24 GO terms and 1 KEGG pathways in the hypothyroid group and 21 GO terms and 2 KEGG pathways in the hyperthyroid group. After PPI network construction, the top five hub genes associated with hypothyroidism were extracted, including ALB, MAPK1, SPP1, PPARG, and MIF, whereas those associated with hyperthyroidism were ALB, FCGR2B, CD44, LCN2, and CD74. The identification of the candidate key genes and enriched signaling pathways provides potential biomarkers for autoimmune thyroid dysfunction related to anti-CTLA-4 therapy and might contribute to the future diagnosis and management of IRAEs for cancer patients.

**Electronic supplementary material:**

The online version of this article (10.1007/s10637-020-00952-z) contains supplementary material, which is available to authorized users.

## Introduction

Immune checkpoint blockade represents one of the most promising anti-tumor immunotherapeutic strategies. Cytotoxic T lymphocyte-associated antigen-4 (CTLA-4), as a key negative regulator of T cell responses, is the first immune checkpoint to be clinically targeted in oncology [1]. CTLA-4 is a type 1 transmembrane glycoprotein of the immunoglobin superfamily and shares 31% amino acid identity with CD28 [2]. It inhibits T cell activity by competing with CD28 for B7 engagement and delivering an inhibitory signal directly from its cytoplasmic tail [3]. Anti-CTLA-4 antibodies, such as ipilimumab and tremelimumab, can exert their antitumor effects via activating CD8 + effector T cells and modulating the function of CD4 + T cells [1]. Ipilimumab alone, or in combination with nivolumab has shown overall survival benefits in patients suffering from metastatic melanoma, metastatic renal cell carcinoma, and other malignancies [4, 5].

However, CTLA-4 inhibition also leads to autoimmune manifestations that are referred to as immune-related adverse events (IRAEs). IRAEs result from the induction of a tolerance break against the tumor and involve a wide range of organ systems [6]. Autoimmune thyroid dysfunction is one of the common IRAEs reported clinically and is described at potentially high risk after checkpoint inhibitor treatment. This includes hypothyroidism and hyperthyroidism, especially after single CTLA-4, PD-1 blockade therapy, or the combination treatment regime [7, 8]. The autoimmune thyroid dysfunction is either caused by primary thyroid gland disorders or as a result of pituitary dysfunction both induced by immune checkpoint blockade [9]. Clinical studies have shown that the incidence of primary and secondary hypothyroidism after administration of ipilimumab was 5.6% and 7.6% of the patients, respectively [9, 10]. However, the time point of primary hypothyroidism occurrence was not clarified. It could be ranging from 5 months to 3 years. Hyperthyroidism is usually found as a transient symptom at the beginning of autoimmune thyroiditis or associated with Graves’ disease after anti-CTLA-4 treatment. Persistent primary hyperthyroidism is significantly less frequent than hypothyroidism with an occurrence of 1.7% [6]. The patients who develop autoimmune thyroiditis after CTLA-4 blockade therapy exhibit symptoms such as pain, hand tremor, periorbital swelling, or tachycardia [11]. These symptoms have a considerable negative influence on the quality of life of patients who are suffering from cancer. Thus, it is critical to identify proper strategies to effectively manage autoimmune thyroid dysfunction after anti-CTLA-4 therapy.

With the advances in high-throughput gene expression profiling technologies, microarrays have become a valuable method to provide insight into the gene expression pattern on a global basis [12]. Gene Expression Omnibus (GEO) database is a repository for high-throughput gene expression data and accessible for the public [13]. Gene data stored in the platform makes it possible to identify differentially expressed genes (DEGs) and find promising biomarkers of human diseases [14]. Another technique that should be emphasized is text mining, also known as Intelligence Text Analysis. Text mining is a powerful tool automatically extracting large amounts of biological information from various written resources by computer [15]. We adopted the public tool pubmed2ensembl to perform text mining. Pubmed2ensembl is an extension of the Ensembl BioMart that integrates the large amounts of genomic data from Ensembl and biological literature from PubMed [16]. This tool allows us to find a set of functionally related genes based on the relevant literature.

The laboratory mouse provides a valuable and accessible model for investigating and elucidating the genetic foundation of human diseases. The rapid development in comparative genetics makes the human and mouse homolog map more detailed and increases our understanding of the high conservation of cellular and metabolic pathways between mouse and human on a molecular and genetic level [17, 18]. Studies have shown that gene expression between the corresponding tissues from different organisms is more similar and conserved than that of the alternative tissues from the identical organism [19]. Mouse gene chips not only provide a complete expression profile of mouse genome but also give us an ideal reference to investigate the underlying mechanism of human diseases on a genetic level.

In this study, we downloaded GSE32445 and GSE58062, the mouse hypothyroidism and hyperthyroidism gene expression profile, respectively, from the GEO database. R language was utilized to standardize and analyze the microarray datasets to obtain DEGs. We then converted the mouse DEGs to their human orthologous genes to deduce the expression pattern of human genes from their mouse orthologues. Text mining about anti-CTLA-4 therapy was then performed by the online tool pubmed2ensembl [16]. After achieving the common genes from microarrays and text mining, the integrated DEGs were subsequently analyzed via GO enrichment and KEGG pathway. The protein-protein interaction (PPI) networks were constructed using the online Search Tool for the Retrieval of Interacting Genes (STRING) and Cytoscape software to identify the candidate hub genes and highly related functional modules.

## Materials and methods

### Microarray data

Gene expression profile matrix files of GSE32445 and GSE58062 based on the Illumina MouseWG-6 v2.0 expression beadchip were downloaded from the GEO database (https://www.ncbi.nlm.nih.gov/geo/). The GSE32445 dataset includes five euthyroid and four hypothyroid mouse liver tissue samples, while the GSE58062 dataset contains four euthyroid and four hyperthyroid mouse liver tissue samples.

### Analysis of DEGs

The downloaded matrix TXT files were normalized with the preprocessCore R package [20]. The DEGs between hypothyroidism or hyperthyroidism and control groups were identified by the empirical Bayes t-test in the Limma R package with |log2 fold change (FC)|>1 and P value < 0.05 set as the threshold values [21]. Then the mouse DEGs were converted to their human orthologs in the HGNC symbol by the Biomart package in R [22, 23].

### Text mining

The public tool pubmed2ensembl (http://pubmed2ensembl.ls.manchester.ac.uk/) was used to perform text mining. The queries “CTLA-4”, “ipilimumab” and “tremelimumab” were retrieved from up to 100,000 document IDs in the dataset of Homo sapiens genes (GRCh37) filtered in MEDLINE. The common genes from DEGs and text mining were then extracted for the following analysis.

### Gene ontology and pathway enrichment analysis

The GO annotation and KEGG pathway enrichment were performed using the Database for Annotation Visualization and Integrated Discovery (DAVID) (version 6.8) (https://david.ncifcrf.gov/) which offers a series of functional annotation tools to analyze the biological meaning of gene lists systematically. The gene lists achieved above were analyzed with a significance threshold of P value < 0.05.

### Integration of PPI network 

We used the online tool STRING (version 11.0) (https://string-db.org/) database to evaluate the relationship between the identified genes above and only interactions with a combined score > 0.4 were considered to be significant. Cytoscape software (version 3.7.2) was used to analyze and visualize the PPI networks acquired from STRING. The Cytoscape plugin Molecular Complex Detection (MCODE) (version 1.5.1) was used to identify the functional modules within the PPI networks with the following default parameters: Degree Cutoff = 2, Node Score Cutoff = 0.2, K-Core = 2 and Max.Depth = 100. Moreover, to explore key proteins in biological networks, another Cytoscape plugin cytoHubba (version 0.1) was applied to identify the hub genes which rank the top five in all nodes based on the topological analysis method “Degree”.

## Results

### Identification of integrated DEGs related to hypothyroidism/hyperthyroidism and anti-CTLA-4 therapy

**Fig. 1:**
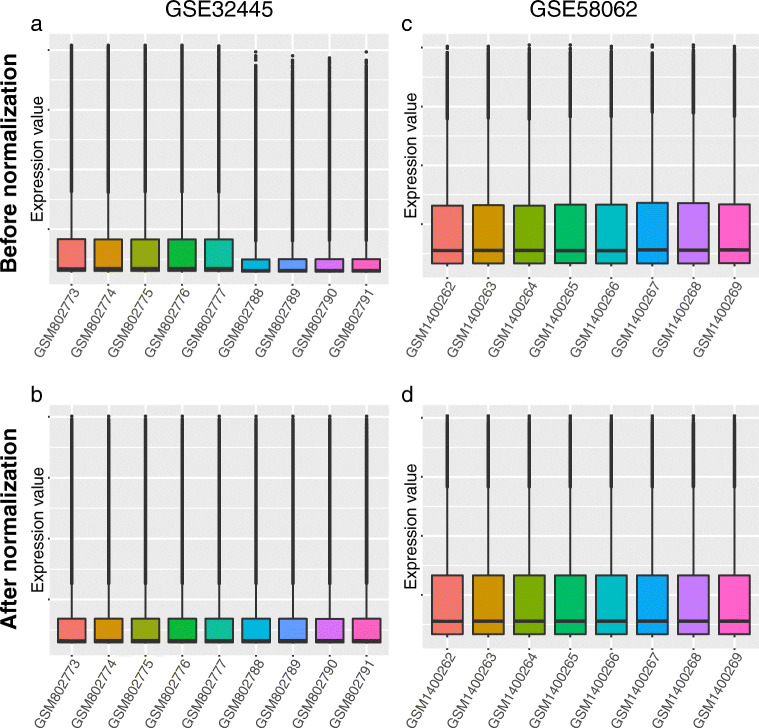
Normalization of gene expression profile matrix. **a**, **b** Before and after normalization of the hypothyroidism GSE32445 dataset. **c**, **d** Before and after normalization of the hyperthyroidism GSE58062 dataset

**Fig. 2:**
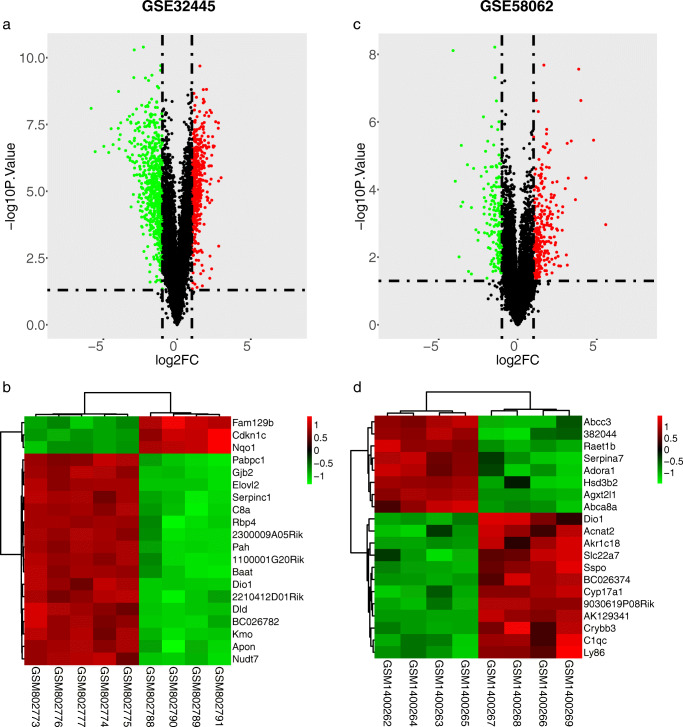
Differentially expressed genes between hypothyroid/hyperthyroid and control groups. **a**, **b** Volcano plot and cluster heat map of the top 20 differentially expressed genes from GSE32445. **c**, **d** Volcano plot and cluster heat map of the top 20 differentially expressed genes from GSE58062. Red represents the upregulated genes based on |log2FC|>1 and P value < 0.05 and green represents the downregulated genes based on the same statistical requirements

Firstly, the hypothyroidism gene expression series GSE32445 and hyperthyroidism gene expression series GSE58062 were normalized, and the results are shown in Fig. 1a-d. After being screened by the limma package, a total of 1270 DEGs between hypothyroid samples and normal controls were identified from the GSE32445 dataset, including 558 upregulated genes and 712 downregulated genes. 475 DEGs between hyperthyroid samples and normal controls were defined from the GSE58062 dataset, including 293 upregulated genes and 182 downregulated genes. The overall distribution and top 100 DEGs of the two datasets are shown in volcano plots and heatmaps, respectively (Fig. 2a-d).

The DEGs acquired from the two mouse microarray datasets were converted to their human orthologs. A total of 948 human DEGs were obtained in the hypothyroidism group, containing 420 upregulated genes and 528 downregulated genes (S.Table 1a). 341 DEGs related to hyperthyroidism were identified and consist of 216 upregulated genes and 125 downregulated genes (S.Table 1b).

**Fig. 3:**
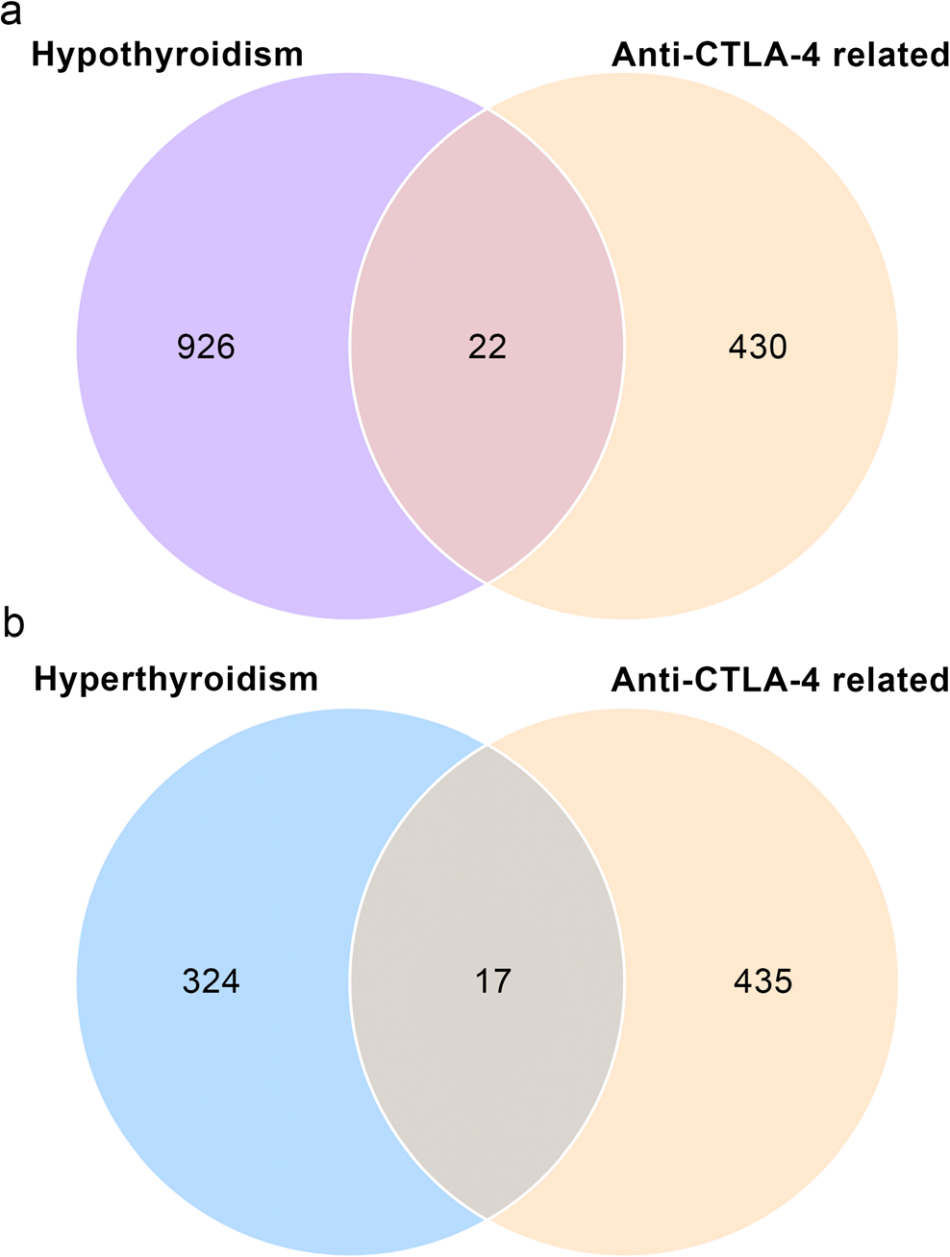
Venn diagram of DEGs from microarray data and genes list from text mining. **a** Intersection of genes between DEGs generated from GSE32445 and anti-CTLA-4 gene list from text mining. **b** Intersection of genes between DEGs generated from GSE58062 and anti-CTLA-4 gene list from text mining. DEGs, differentially expressed genes

452 human genes associated with anti-CTLA-4 therapy were revealed by text mining (S.Table 1). After comparing the DEGs from microarray data to the gene list derived from text mining, the intersections of genes were obtained and involved 22 genes in the hypothyroidism group and 17 genes in the hyperthyroidism group (Fig. 3).

### Functional enrichment analysis

**Fig. 4:**
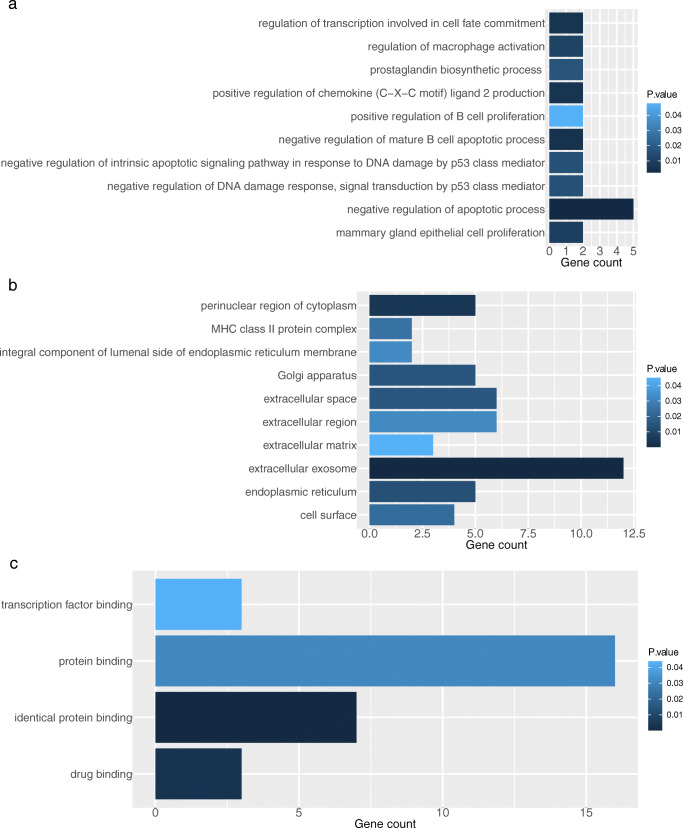
GO analysis of common genes associated with hyperthyroidism and anti-CTLA-4 therapy. **a** biological process. **b** cell component. **c** molecular function

**Fig. 5:**
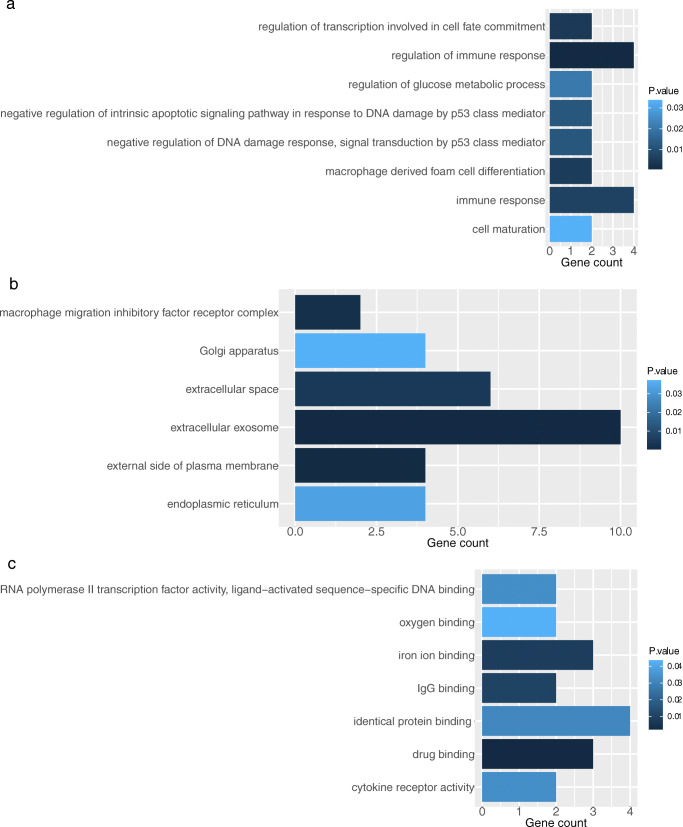
GO analysis of common genes associated with hyperthyroidism and anti-CTLA-4 therapy. **a** biological process. **b** cell component. **c** molecular function

DAVID was utilized to perform GO and KEGG enrichment analysis based on the integrated DEGs achieved above. This was done to investigate the correlated biological function of the integrated DEGs in autoimmune thyroid dysfunction related to anti-CTLA-4 therapy. In the results of GO analysis, 10 biological process (BP) terms, 10 cell component (CC) terms, and 4 molecular function (MF) terms were identified in the integrated DEGs of hypothyroidism with a P value < 0.05 as the significant threshold. 5 genes were mainly enriched in the BP term “negative regulation of apoptotic process”, 12 genes fell into in the CC term “extracellular exosome” and 7 genes were involved in the MF term “identical protein binding” (Fig. 4 and S.Table 3a). In hyperthyroidism, integrated DEGs were significantly enriched in 21 GO terms including 8 BP terms, 6 CC terms and, 7 MF terms. The genes were primarily enriched in the following terms: “regulation of immune response” in BP, “extracellular exosome” in CC, and “drug binding” in MF. These were the top 3 terms of GO annotation with the integrated genes enriched most significantly (Fig. 5 and S. Table 3b).

**Fig. 6:**
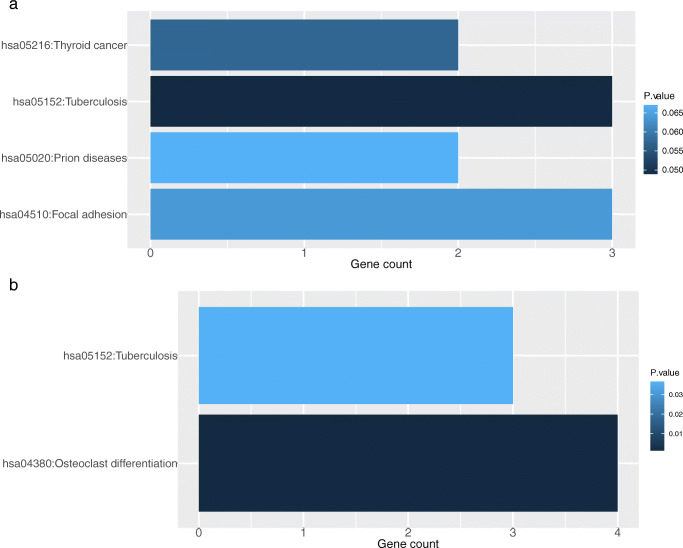
KEGG pathway enrichment of the integrated DEGs associated with autoimmune thyroid dysfunction and anti-CTLA-4 therapy. **a** hypothyroidism. **b** hyperthyroidism. DEGs, differentially expressed genes

The KEGG enrichment analysis revealed that the integrated DEGs were significantly enriched in the KEGG pathway “tuberculosis” in hypothyroidism group and “osteoclast differentiation”, “tuberculosis” in the hyperthyroidism group (Fig. 6 and S.Table 4). Notably, even though the other three pathways “thyroid cancer”, “focal adhesion” and “prion diseases” in hypothyroidism were not considered significant, the gene MAPK1 was enriched in all the hypothyroidism related KEGG pathways.

### Construction and module analysis of PPI network

**Fig. 7:**
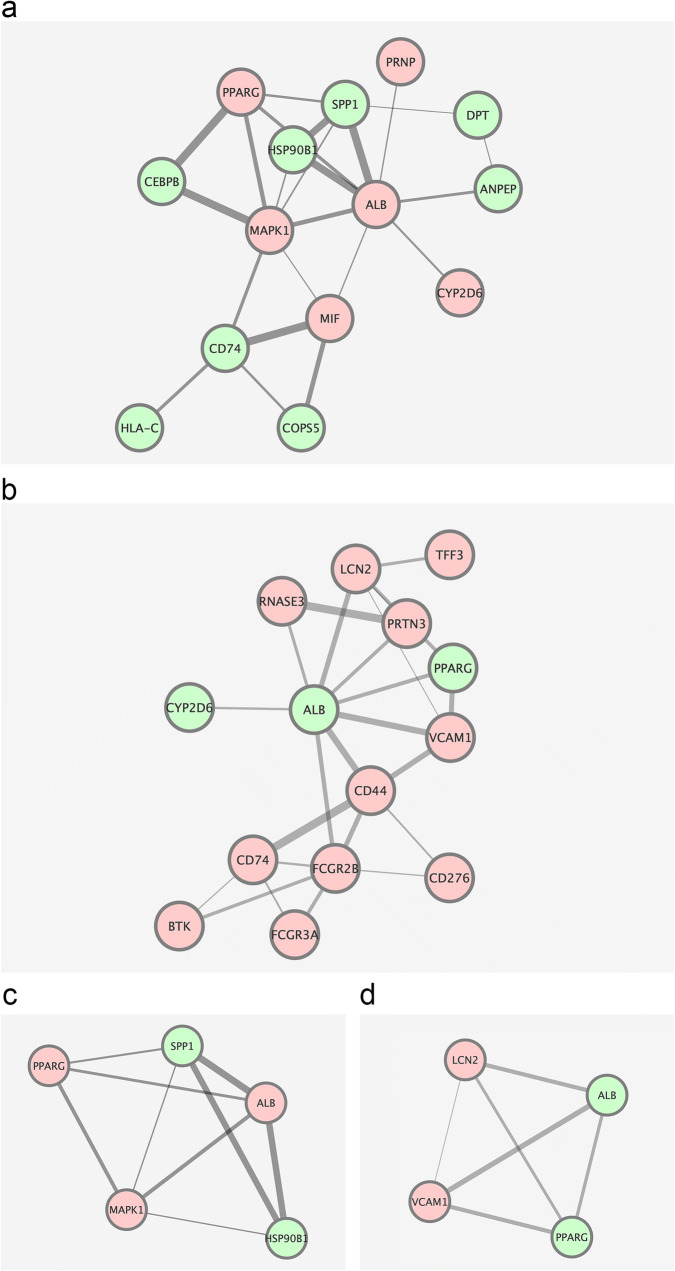
PPI network and highly connected modules of integrated genes. **a**, **b** PPI network in hypothyroidism and hyperthyroidism group. **c**, **d **Modules from the PPI network in hypothyroidism and hyperthyroidism group generatedby the MCODE algorithm in Cytoscape. Red indicates relative upregulated genes and green indicates relative downregulated genes. The size of the edges represents the strength of the interactions based on the combined score

The STRING database and Cytoscape platform were adopted to perform PPI network generation, module analysis, and visualization based on the 22 genes in the hypothyroidism group and 17 genes in the hyperthyroidism group. A PPI network containing 23 interactions was generated based on 14 integrated DEGs correlated with hypothyroidism (Fig. 7a). Also, the PPI network in the hyperthyroid group was constructed and contained 14 connected DEGs (Fig. 7b). According to the degree value, the top five hub genes extracted from the hypothyroidism group were ALB (albumin), MARK1 (microtubule affinity regulating kinase 1), SPP1 (secreted phosphoprotein 1), PPARG (peroxisome-proliferator activated receptor gamma) and MIF (macrophage migration inhibitory factor). On the other hand, in the hyperthyroid group, the top five hub genes were ALB, FCGR2B (Fc fragment of IgG receptor IIb), CD44, LCN2 (lipocalin 2), and CD47 (Table 1).

**Table 1:** Top five hub genes identified from the PPI networks

Hypothyroidism related genes		Hyperthyroidism related genes	
Gene	Node	Gene	Node
ALB	8	ALB	8
MAPK1	7	FCGR2B	6
SPP1	5	CD44	5
PPARG	4	LCN2	5
MIF	4	CD74	4

MCODE algorithm was performed to detect highly interconnected subnets that are usually protein complexes and parts of pathways based on the topological structure. One highly connected cluster was constructed with 5 nodes and 9 edges from the PPI network of hypothyroidism, including 3 upregulated and 2 downregulated genes (Fig. 7c). A cluster from the network of hyperthyroidism was generated with 4 nodes and 6 edges, containing 2 upregulated and 2 downregulated genes (Fig. 7d). Further functional enrichment analysis of the identified modules revealed that genes in the module of hypothyroidism were primarily enriched in the GO terms of “response to stress”, “perinuclear region of cytoplasm”, “identical protein binding” and KEGG pathway of “PI3K-Akt signaling pathway” (Table 2a). Genes in the module of hyperthyroidism mainly fell into the GO terms of “response to nutrient”, “Golgi apparatus” and “drug binding” (Table 2b).

**Table 2:** Functional enrichment analysis of genes from the highly interconnected modules. P value<0.05

Category	Term	ID	Count	P value
a. Hypothyroidism				
BP	Response to stress	GO:0006950	2	0.014453026
BP	Response to estrogen	GO:0043627	2	0.01539526
BP	Receptor-mediated endocytosis	GO:0006898	2	0.043579902
CC	Perinuclear region of cytoplasm	GO:0048471	3	0.006645122
CC	Golgi apparatus	GO:0005794	3	0.012608444
CC	Extracellular exosome	GO:0070062	4	0.012971117
CC	Extracellular region	GO:0005576	3	0.041477897
MF	Identical protein binding	GO:0042802	3	0.011112225
MF	Protein phosphatase binding	GO:0019903	2	0.014845978
MF	Drug binding	GO:0008144	2	0.017888742
KEGG	PI3K-Akt signaling pathway	hsa04151	3	0.007274867
KEGG	Pathways in cancer	hsa05200	3	0.009397898
KEGG	Thyroid cancer	hsa05216	2	0.012595768
KEGG	Prostate cancer	hsa05215	2	0.037894255
KEGG	Estrogen signaling pathway	hsa04915	2	0.042562603
KEGG	Toll-like receptor signaling pathway	hsa04620	2	0.045525493
b. Hyperthyroidism				
BP	Response to nutrient	GO:0007584	2	0.013163186
BP	Cellular response to tumor necrosis factor	GO:0071356	2	0.019524915
CC	Golgi apparatus	GO:0005794	3	0.006508414
CC	Extracellular space	GO:0005615	3	0.015572412
MF	Drug binding	GO:0008144	2	0.013446386

## Discussion

IRAEs occur in approximately 60% of the patients after ipilimumab administration [5]. They can potentially involve every organ system, but the gastrointestinal tract, skin, liver, and endocrine glands are more commonly affected [24]. When the combination of CTLA-4 and PD-1 blockades are adopted, the symptoms are more severe and can be fatal in some cases [25, 26]. Even though IRAEs are frequently described, the optimal treatment strategies have not been defined and primarily depend on consensus opinion [27].

Autoimmune thyroid dysfunction related to CTLA-4 antibody therapy is one of the common IRAEs clinically. A single-center study reported that a total of 23% of patients with melanoma treated with ipilimumab developed different degrees of thyroid abnormalities, including primary or sub-clinical hypothyroidism and hyperthyroidism [8]. It is crucial to discriminate between primary and secondary thyroid dysfunction and exclude the pre-existing thyroid disorders for the proper treatment. Thus, the thyroid function of patients before and after anti-CTLA-4 antibody treatment should be assessed routinely. Despite its importance, scheduled thyroid laboratory evaluation is often complicated and inaccessible in cancer patients. This is due to the fact that both, acute illnesses and antitumor therapy, can affect triiodothyronine (T3) levels in patients [28]. Furthermore, repeating evaluation and following up of thyroid function also becomes a burden for both patients and medical resources. Therefore, it is critical to elucidate the molecular mechanism of the autoimmune thyroid dysfunction after anti-CTLA-4 therapy in order to find effective biological markers and efficient strategies for the diagnosis, monitoring, and treatment of patients.

In this study, 22 and 17 genes in the hypothyroidism and hyperthyroidism group, respectively, associated with anti-CTLA-4 therapy were identified for functional analysis by GO and KEGG enrichment. Results from GO annotation suggest that these DEGs are mainly involved in apoptosis, protein binding and immune regulation. It should also be noted that MAPK1 is the common gene in all the enriched KEGG pathways in the hypothyroidism group and is one of the hub genes identified from the PPI network. MAPK1, also known as mitogen-activated protein kinase 1, is a member of the MAP kinase family and is involved in a wide range of biological processes like cell proliferation and angiogenesis. Thyroid hormone activates MAPK by binding to the hormone receptor ανβ3 integrin expressed on various tumor cells to promote cell proliferation. A study demonstrated that an inhibitory thyroxine (T4) analogue tetraiodothyroacetic acid (tetrac) blocked MAPK activation induced by thyroid hormone and prevented the proliferation of myeloma cells [29].

The common genes enriched in two KEGG terms of the hyperthyroid group were FCGR2B and FCGR3A. FCGR2B, also named CD32, and FCGR3A, also named CD16A, are low-affinity receptors for the Fc region of immunoglobulin gamma (IgG) belonging to the immunoglobulin superfamily and involved in a series of immune response. FCGR2B is the only inhibitory IgG Fc receptor that prevents the immune overstimulation and regulates immunologic balance [30]. FCGR2B expressing on B cells suppresses humoral immunity by restraining their activation and inhibiting B cell-mediated antigen presentation to T cells [31]. Therefore, the dysfunction of FCGR2B affects the susceptibility to several autoimmune diseases. The activating Fc receptor FCGR3A is mainly expressed in NK cells and macrophages and plays an important role in autoimmune disease [32]. A previous study indicated that the hypothyroidism due to rabbit immunoglobulins injection was attenuated in mice lacking FCGR3, but not in mice lacking FCGR2B [33].

By the PPI network construction, functional enrichment analysis of the highly connected modules revealed that genes in the hypothyroidism module were mainly enriched in the KEGG term “PI3K-Akt signaling pathway”. Phosphatidylinositol 3′ -kinase (PI3K) is responsible for the generation of phosphatidylinositol 3,4,5-trisphosphate, a second messenger essential for the translocation of Akt (Protein kinase B). Activation of Akt involves in fundamental cellular functions such as cell proliferation and survival. The PI3K-Akt pathway is associated with the development of various diseases such as cancer, diabetes mellitus, and autoimmunity [34]. Furthermore, five hub genes with the highest degree of connectivity were identified separately from the hypothyroidism and hyperthyroidism group. The top five hub genes associated with hypothyroidism are ALB, MAPK1, SPP1, PPARG, and MIF, whereas those associated with hyperthyroidism are ALB, FCGR2B, CD44, LCN2, and CD74.

Specifically, the hypothyroidism and hyperthyroidism group share the same hub gene ALB, also known as albumin. Albumin is a multifunctional plasma protein that is highly abundant in human blood [35]. The center of Albumin consists of hydrophobic radicals which are binding sites for a wide range of compounds, such as the hormone T4. It serves as a critical fast exchange resource of the thyroid hormone to rapidly stabilize T4 level [36]. A total of four binding sites for T4 have been identified on the subdomains IIA, IIIA, and IIIB of human serum albumin [37]. The mutation of residue R218 in its subdomain IIA is related to familial dysalbuminemic hyperthyroxinemia (FDH) which is induced by the increased affinity for thyroxine after conformation change and the further elevated serum T4 levels [38].

SPP1, also named osteopontin, plays an important role in cell-mediated immunity and immunoglobulin production by B cells [39, 40]. It is involved in various autoimmune diseases including systemic lupus erythematosus, rheumatoid arthritis, and multiple sclerosis [41]. It was demonstrated that SPP1 levels were increased in the serum of patients suffering from Graves’ disease [42].

PPARG encodes a member of the peroxisome proliferator-activated receptor (PPAR) subfamily. There are three known isotypes: PPARα, PPARβ, and PPARγ. PPARγ is primarily expressed in adipose tissue, colon and immune system and takes part in distinct diseases including obesity, diabetes, atherosclerosis and cancer [43]. A previous study indicated that the PPARγ agonist rosiglitazone raised the thyroxine hormone levels in circulation and PPARs and thyroid hormone receptors could crosstalk to influence a series of biological processes [44, 45].

MIF is a lymphokine released by macrophages, lymphocytes and epithelial cells and involved in a variety of cell-mediated immune response and inflammation. T4 is considered as a potential antagonist of MIF as the binding of T4 suppresses the catalytic activity of the hydrophobic pocket that is associated with the pro-inflammatory activities of MIF. In patients with active antineutrophil cytoplasmic antibody (ANCA)-associated vasculitis (AAV), an increase in MIF was detected with a decreased fT3 level [46]. It is noteworthy that MIF binds to its natural receptor CD74, a type II transmembrane protein, to initiate downstream inflammatory signals. The recruitment of CD44, a transmembrane glycoprotein with tyrosine kinase activity, is necessary in the following activation of SRC family non-receptor tyrosine kinases, which result in the phosphorylation of ERK1 and ERK2 [47, 48]. In addition, a study about Graves’ disease (GD) showed that the injection of GD lymphocytes into thyroid tissue transplants increased the expression of CD44 significantly, indicating the important role of CD44 in the pathology of autoimmune thyroid diseases [49].

LCN2 is a small extracellular protein belonging to the lipocalin family. Besides its transportation function of hydrophobic molecules such as lipids, steroids, and retinoids, LCN2 is also associated with immune modulation, metabolic regulation, and tumorigenesis [50]. LCN2 was indicated to be regulated by the thyroid hormone. A study showed that thyroid hormone receptors bound directly to thyroid hormone response elements that are located between positions − 1444 and − 1427 of the LCN2 promotor and T3 upregulated the expression of LCN2 in a time- and dose-dependent way [51].

## Conclusions

As one of the relatively common IRAEs, autoimmune thyroid dysfunction after anti-CTLA-4 therapy should be brought to the forefront, especially in the systematic therapeutic regime of malignancies in clinical practice. In the present study, the pivotal role of candidate key genes such as MAPK1 and FCGR2B, and the enriched signaling pathway such as PI3K-Akt pathway in the molecular regulation network of autoimmune thyroid disorders were highlighted by integrated bioinformatic analysis and this provided potential targets for the future diagnosis and clinical treatment. Nevertheless, in vitro or in vivo experiments should be performed for further verification of the obtained findings.

## Electronic supplementary material

ESM 1 (PDF 186 kb)
