# Supplementary material for: Integrative analysis of key candidate genes and signaling pathways in autoimmune thyroid dysfunction related to anti-CTLA-4 therapy by bioinformatics
Source: Invest New Drugs. 2020 Jun 4;38(6):1717–29. doi: 10.1007/s10637-020-00952-z (PMC7575511; doi:10.1007/s10637-020-00952-z)
Supplement: Supplementary file 1 — (PDF 186 kb) [file 10637_2020_952_MOESM1_ESM.pdf]

# Supplementary data:

**Article title:** Integrative analysis of key candidate genes and signaling pathways in autoimmune thyroid dysfunction related to anti-CTLA-4 therapy by bioinformatics

**Journal name:** Investigational New Drugs

**Author names:** Ying Zhang, Francesca Garofano, Xiaolong Wu, Matthias Schmid, Peter Krawitz, Markus Essler and Ingo G.H. Schmidt-Wolf

**Correspondence to:** Ingo G.H. Schmidt-Wolf, Department of Integrated Oncology, University Hospital of Bonn, Venusberg-Campus 1, D-53127 Bonn, Germany. Tel.: +49 22828717050, Fax: +49 228 28717065, e-mail: ingo.schmidt-wolf@ukbonn.de

**Supplementary Table 1** Differentially expressed human genes identified from the hypothyroid and hyperthyroid samples compared with their normal controls. P value<0.05

a. Hypothyroidism

| DEGs | Gene list |
|---|---|
| Upregulated | MBD3L5 PIGT MCM10 BAG1 MYO9B SNRPD1 CCDC127 MAN2C1 EXOSC7 USP19 PTDSS2 PTBP1 CDS2 HP1BP3 PPFIBP2 EIF2B2 OLFML1 SLC35B3 EXTL3 HARS CISH MIF GINS1 SESN1 HEMK1 TMEM131 L3MBTL3 GNPTG TRIB3 DNMT3B PTS ZFAND2A C3orf18 ZKSCAN5 ACYP1 MIDN ERAL1 HDC MORC2 TMEM51 ZFAND1 POLE3 GSTT2B THNSL2 ACTR1A GFER PPARG FLOT2 SCUBE3 MRAP PARK7 IMP4 TBCEL LEMD2 FIBP ACAD8 NUP210 RHBDF1 POLDIP2 GGNBP2 NT5C3A RASGRP2 BRD9 IREB2 APLP2 STUB1 EPAS1 ATG2B CHST8 DECR1 RASA3 SHROOM2 UBOX5 DYM C3orf14 SPSB2 RASGRP4 PTTG1IP INPP5K IFT172 C20orf27 PIPOX EIF2AK2 SLC25A28 CLDN9 CNNM2 NEDD4L MAPK1 ITPR1 CUTC RAC1 MT1F OR5AN1 REC8 MIB2 LSM4 CRELD1 ZYX ALKBH7 GTF2F1 DARS AQP9 JAGN1 SLC44A1 OTUD3 TMEM184B COX6B1 STAB1 DCT ADARB1 MAGED2 RENBP ANTKMT PPM1F SLC25A37 ADAM23 PCBP4 RBBP7 ZBTB17 FARS2 GPR1 CEBPE IRF2BP1 AKR7L UPP1 LUC7L DUS4L ACYP2 FGFRL1 UACA KRTCAP3 ATP6V1C2 LYPLA2 CHMP4B SMN2 ATAD3A PLOD3 WFDC2 SAE1 HYAL2 PGLS C19orf44 ZNF330 ANKH FLOT1 IER3 RASSF1 DNTTIP1 OLIG1 CHCHD4 ABCC4 UBAP2 CYP2A7 SERGEF CSGALNACT2 MMP14 SCARF2 MECR INTS4 OVOL2 SAP30L CIRBP CDCA4 TTYH2 RNF6 PXT1 SEMA4B NRM C8orf82 RNPEPL1 DYNLL2 SREBF2 AQP8 ASB1 BLVRB GAMT GALM COMMD6 SCAMP5 OC90 ADSSL1 GCKR GALK1 DDX17 TSTA3 AKAP8L SERPINB1 NR1I3 ACOT1 DENND5B PPEF2 MNAT1 CSNK1G2 NQO1 LRBA ATP5IF1 SGK1 POP4 MGST3 CYP2D6 TMEM38B IPO5 DENND3 HEBP1 TBC1D5 CSTF1 ETHE1 PUS1 CA14 PGS1 APPL1 UGCG DNAJC13 IL11RA SLC25A19 UBXN6 EIF6 PPME1 SERPINA7 PRNP RPL22 CRYL1 GSTM1 GTPBP2 LRTM1 ERP29 PHC2 PSPC1 IL6R CLN3 HEATR1 |

| | |
|---|---|
| | MRPL38 MRPS34 NIT1 ATOX1 NSMCE1 MRPS9 GTF2H3 CDIPT C1orf198 FBXO6 ISY1 TIAM2 PPP3R1 OR5AP2 CNGA1 NRBP2 PFDN2 C9orf78 NEU2 LMBR1L REEP4 ZNF623 C11orf80 PRKD2 ABHD10 CLEC11A SPON2 NIBAN2 DBP TMEM8A ISOC2 CBR3 TMCO6 RABGEF1 ZFYVE21 CDKN1C LLGL1 SLITRK1 SLC2A2 TIMM50 ACVR2B SH3GLB2 NRBF2 RAB21 MPST PTRH1 CTF1 GARS ZFHX2 OR11H7 VPS37B RDH5 SOAT2 KRT23 SNX17 MSRB2 GPR45 SFXN5 MFGE8 TNNT1 DYNC1H1 PAK4 ZER1 BOLA1 MAEA PIH1D1 HTATIP2 RAP1GAP TBC1D22A ARRDC4 AXIN1 POFUT1 KCNJ3 ABCG8 RSAD1 LRRC59 TXNL4B RETSAT KLF16 CYP4A11 EEF1G ALG9 MVP PI4KA C6orf226 SCAMP2 TRAPPC10 LPL SF3B2 PRRC1 EIF3D PLEKHA4 NUDT19 LRRC46 SNX2 TUT1 MGRN1 WDR13 GPC1 DOCK6 FMO1 ALB BRF2 CTNNAL1 MTSS1 DBNDD2 MTCH1 UBE2E2 UAP1L1 PARD6B APOA4 APEH CSNK1D NRF1 PIP5K1A FFAR3 VAMP2 TCEAL8 PGAM5 SLC6A9 PFN2 RIT1 ABCC3 UGP2 DDHD2 PSMD4 SPG21 ABTB1 DIABLO SLC1A7 FNTB PROX1 RFC5 EPHX1 TMED4 SEMA4A IL4I1 DDIT3 SLC35E3 FBXW5 EDNRA C16orf58 ADHFE1 GIMAP6 ZDHHC7 UBE2E1 PRDX6 SPOP NIPSNAP1 KPNA6 CWF19L2 DIS3L2 KLHL26 UGT1A6 FAM20B INHBE TEX2 SCAMP1 APOA5 GPBP1L1 CMTM4 C18orf21 HPD EIF3I CC2D1A MYLK CPEB3 ZMYM3 ADORA1 GBA SEMA3F PKP4 HCN3 CDC42SE1 TSC22D3 RASL11B STXBP5L CLDN5 DCUN1D4 LSM1 GTF2E1 RGS16 PEX11B FBXO28 BAG4 CYP4F8 PPP1R3C CHRD CUTA |
| Downregulated | KLHL7 RGN TSG101 RPS21 VPS26B KIF1B MTMR14 SIDT2 ASNSD1 HUWE1 ARID4A ALDH1B1 TRAPPC6B IL6ST TFF3 ATP5F1D CTSB TRIM56 NKIRAS1 RNASEK SMPDL3A TRAK1 IFT20 STARD10 SKIV2L ADCY9 GPR146 EED ZKSCAN4 SLC25A42 PON3 PMPCB IDI1 DHCR7 CHMP5 YTHDF2 CNOT4 COX6C COPS5 COMT ZNF263 PSMC2 FOS ELOVL3 ACADSB UBL3 CYP7A1 USP4 CHUK RPL18A FNIP1 HAO1 IRF9 DEK CUEDC2 C2CD2L SPAST HSD17B12 PUS10 PPP1R3B NFIC KLB C4A BCAP31 BCL2L13 MRPL36 GNAI2 MEMO1 KRCC1 CRIPT DDT NARS SRPRA SEC11C WDR82 MFHAS1 LMAN1 VAMP8 ANG TUBB6 ZC3H7A LHFPL6 PURB EIF1B AFF1 SLC25A30 GSTK1 TLCD2 PRPF8 DUSP1 TAX1BP1 |

IFITM2 CYP3A5 RPUSD3 ARPC2 PANK3 PECR TCP1 CRLS1 C1QC SEC24D CALM2 RGS10 ZMAT1 ARL3 MRPL52 PPARD SKP1 CHD7 IGFBP1 GMPS HINT2 ETFA YWHAG CYP2F1 HCFC1 SPCS1 PAIP2 TOMM70 CTSC MDH2 LYVE1 SLC1A2 NSFL1C GDF15 CD74 HMGCR YWHAB IFRD2 ALDH1A1 PCDH1 TM7SF2 ELAVL1 MPND SNX14 PDIA6 PBRM1 DAAM1 U2AF2 COLEC10 SNX1 MRPL43 SCOC HSF2 COLEC11 ABI1 CPB2 SCFD1 MTTP TUSC1 DHRS7 GPC4 NUDCD2 SPP1 AZGP1 PSMD14 PRKD3 HLA-C C8A YTHDF1 EIF4EBP2 CMTM6 C20orf194 C5orf24 PYGL MPV17 SNRPD3 CYP17A1 NPR2 SLC38A4 NPC1 PPIB H2AFY RGP1 SEMA4G PSMD11 COX7A2 DAG1 TXNDC15 PCMTD2 CALD1 PLAC8 SERINC1 RBP4 REXO2 PGRMC1 RAD23B HAMP ANKRD46 MRPS18C NUCB1 COX11 ERGIC2 DUSP6 DDX1 ZNF292 PRDX5 YIPF2 EDEM2 SERPINA3 CLDND1 UBE2A TMOD3 PAPSS2 CDH1 SEC63 RNF11 GRB14 VAPA SLC66A2 MAD2L2 SNX3 MRPS2 GLRX3 CCNG2 MAGOH KLF15 GJB2 ACBD4 PCK1 ANGPTL3 DDC IRGM ACSL1 UHRF2 S100A13 COMMD1 AGPAT2 ZCCHC17 TFAM ZC3H15 ACTL6A PLS3 KRT8 SAA2 PTCD3 UCHL3 IMMT ABCD3 RRN3 NDUFB4 BCKDHB EMG1 UBFD1 CS SPARC PFN1 NFU1 PPP1CB GLS2 ATXN10 TMEM19 TMEM167B CCNL1 IGFBP2 EXOC7 ESD DPYD AHSA1 EGR1 PMM2 HSP90AB1 ELOVL2 BUD31 TMEM59 DDOST DDX3X RIC8B ALDOB PTPRB HABP2 PLXNB2 DEXI PSMD6 CFDP1 NFIB TOR1B ZFHX3 VDAC2 ID2 STK40 TMCC1 AADAT GSTM3 NDRG2 ZMPSTE24 TSPAN12 DLD MBD1 POLR1B SERPINA12 PCMT1 ATP5F1A EIF3K IFI27L2 GOSR1 CREB3L3 GMCL2 METTL7B TWISTNB TMSB4Y PCYT1A HYOU1 GCLC NDUFA1 KPNA3 CYP20A1 TIMM10 ARNTL PEX7 MCL1 NDUFB9 COX5A DPT PHAX FTSJ3 HMOX1 FLNB GDE1 C5orf34 DNAJB9 SLAIN2 DUSP16 SP1 BMPR1A NDUFB5 SAA4 ZHX2 SH3GLB1 NDUFS3 GNMT DIO1 SH3BGRL2 LGALS13 HADHB NME7 R3HDM2 CEACAM4 CDV3 LPCAT3 CTSZ HACL1 CHMP2A SLC25A25 KMO DUSP23 PRODH ALCAM ZFP36L1 MRPL16 SH3BGRL NOSTRIN ABCB7 TAF13 FNDC3B USP10 RAB5C CUL1 CBR1 PNO1 MRPS35 HMOX2 LY86 PLSCR1 PPM1K KPNB1 APOF COG2 HES6 ATL2 LONP2 PREPL C6 ATP5F1E GLTP DCN NUDT7 LRRC8A AGPAT3 CCT8 FOXQ1 ETFDH ARL8A CCDC25 AASS ITIH2 IRF2BP2 NDST1 SLC9A3R1

| | |
|---|---|
| | ACO1 NDFIP2 POLR2G YME1L1 OSBPL2 DCUN1D5 SNX7 BAAT HIST1H2BE ADAM9 VBP1 MBL2 SLC27A2 FASTKD1 C1GALT1C1 GFM1 TP53INP2 AKR1E2 PPP4R1 ATP2A2 CFB NMD3 PDCD5 B3GALT1 KIF1C RALBP1 PA2G4 ZFYVE1 MINPP1 CRELD2 EHHADH MAP1LC3B2 TSN BCL6 ATP5PB ATF5 NAGLU SUSD4 SEC16B BCAS2 TDO2 ACP6 FAM162A AADAC ELP2 FGFR1OP2 CTSS KPNA1 RASSF3 CCDC6 TOMM7 SLC35B1 SS18 PIGR TGIF1 AVPR1A KNG1 ARCN1 C11orf54 PSME3 ACADL NR2C1 PSMD12 ULK2 RCHY1 EFNA1 CMAS PTPRE TXNDC12 ZBTB43 MON1A GSTP1 MASP1 PTPRD SARDH SERPINC1 CLDN12 SEPHS2 MGST1 AFM IDH2 C15orf61 SRP9 SDHD CHCHD7 RAB7A MCCC1 SLC25A17 MGLL CGN PSMD9 CEBPB TPCN1 CITED2 AK3 ATXN1 OSGIN1 UHRF1BP1L ACY3 CDO1 APOH MRPL22 PAH LMF1 HAL S100A10 HSPA5 SELENBP1 PTGES3 VTI1B HSP90B1 GLUL CPN2 MLYCD ZNF750 UPP2 ATP6V1A PHLDA1 CYP7B1 TCF25 TM7SF3 LSM6 LIMA1 EI24 DAZAP2 LPGAT1 COX7B LARP4 PDPK1 MED28 HSD17B2 ITGB5 CAPRIN1 USP33 ANPEP INHBC ST13 HSD17B6 RP9 |

b. Hyperthyroidism

| DEGs | Gene list |
|---|---|
| Upregulated | HIST1H4J TSKU TFF3 CCL24 TNFAIP2 ALDH3B1 SQLE CD44 CSF2RA MYOM3 STIM2 CRYBB3 C1QC STX1A LRFN3 NSDHL ALOX5AP TLCD2 FZD7 TYROBP TBXAS1 SLC1A3 SLC25A30 RRM2 GPX1 RSPH1 MAD1L1 C1QA FOXO1 VSIG4 C1QB ASB2 TUBB6 ENO1 HK3 HIST1H1C NCAPH FOLR2 ANKS1A PLD4 RNASE3 KIRREL3 LGMN HOPX CD276 PRC1 SDC3 MCM6 CTSC ST3GAL5 PPARD DNAI1 AIF1 HIST1H2AB CFP CALM2 HLA-DMA HIST1H3A ISYNA1 PRTN3 NT5E LAPTM5 SIGLEC1 FGL1 CD74 COL12A1 GDF15 SLC7A8 P2RY6 SLC22A7 SDF2L1 EVL CYP17A1 PLVAP PLAC8 MTHFD2 PSAT1 APCS KCNK1 HYOU1 CHTF18 SSPO PLA2G7 HMOX1 HP CDCA7 KCNK13 LY86 PBK COL15A1 CBR1 HIST1H4I PRKCB CLEC4A TNK2 NNMT VCAM1 SLC25A45 DIO1 TROAP PTGS1 FNDC3B FNDC5 FCGR1A SLC15A3 IDH3A PTGIS EMILIN2 SORT1 B3GALT1 CIB2 MMD2 ZNF467 APOM WAS AURKA CPNE2 NUDT18 COTL1 ACOT11 TOMM40L TUBA1B PLK1 PFKFB4 FIGNL1 SAA2 LCN2 LGALS13 |

| | |
|---|---|
| | SLAMF9 LYZ CUX2 ANTXR2 FGF21 RAB34 CDCA2 SELPLG FCGR2B A1BG GBP3 REEP5 DCN MGP AXL INCENP SLC13A3 MS4A8 TLE6 SULT1A4 NCKAP1L PCOLCE2 INSIG2 MCM5 GLTP CDKN1A EGR1 CLEC4F PDLIM4 NCF4 ANLN SNX10 CXCL14 CD52 CLDN14 PAQR9 PREPL FCGR3A IFI27L2 MS4A6A SEC61B CD207 WFDC12 NANS PNPLA3 NMRAL1 LPL ADRB2 CKB TMPRSS2 TP53INP2 CYP51A1 LGALS3 CDKN3 S100A9 SPP2 CDC20 SLC41A2 CYBA CD53 PGK1 MVD CLEC7A SOAT1 TRHDE WFIKKN1 PIK3AP1 CYP7B1 BCL3 SLC16A6 FPR2 SNAI3 HSD17B2 ARHGAP24 GNGT2 C19orf67 S100A8 BTK KIF23 KIF2C ITGB1BP1 AATK P2RY13 BMP7 INSC TIMD4 ATXN1 RAD54L |
| Downregulated | MACROD1 MCM10 DQX1 ZNF395 SOX12 CCT6B MARVELD1 TRIB3 ADCY9 VWCE RANBP3L SMARCA2 PDE4C SLC13A4 OAT HDAC11 TCEA3 PPARG LCMT2 TMEM201 PPL SLC25A37 APOL4 C20orf27 SLC44A1 VLDLR COL27A1 GPR135 OLIG1 UPB1 NOX4 MRPS26 PCBP4 CEBPE MRPL18 KRT23 MGST3 AQP8 CABYR BRAP KIFC2 GAL3ST1 IL17RB IGFBP5 LONRF3 CACNA1D ACOT4 ELL3 ACVR2B DOCK6 COL20A1 KRT8 BHLHB9 ADSSL1 KISS1 ADCY6 NPFF ENPP2 EGLN3 SLC6A16 RAP1GAP LIMD2 OCIAD2 CISD1 SLC2A9 ALB ARSA CYP2J2 RNASEH2A RABEP2 ACOT1 BDH2 ADAMTS7 NGEF HOOK2 NR1I3 SPSB4 CHDH HAMP PCCB SERPINA7 MAPK15 CYP2D6 SLC2A2 C19orf12 MKNK2 LGALS4 CDKN1C C4orf47 SLC23A3 BUD13 CLCN2 RDH16 FAM13A C12orf43 ETNK2 CCDC57 NFYB ZMYM3 EPHX1 C16orf74 CYP4F2 TMEM25 ABCC3 AQP4 PDK1 SLCO1A2 DEGS1 ADORA1 SLC17A8 RORC ECHDC2 SUCNR1 LINGO4 TOB1 ROBO1 GRIK5 DCLK3 SARDH HSD17B6 INHBE PRSS8 HSD3B2 SORBS3 SPATA2L |

**Supplementary Table 2** Detailed human gene list related to anti-CTLA-4 therapy retrieved from text mining

| | Gene list |
|---|---|
| Anti-CTLA-4 related | LYST LBR IRF6 CD34 IL10 PDCD1 LAD1 PTPRC CFH PTGS2 SAG LAMC2 NPL SOAT1 TNFSF4 TNFSF18 FASLG AGFG1 SELL F5 SELP XCL1 DPT CD247 PTPRN FCGR3B FCGR3A FCGR2A CD48 SLC11A1 MNDA FCRL3 LMNA MUC1 ICOS CTLA4 CD28 ZBTB7B SHC1 CFLAR TCHH RORC HSPE1 HSPD1P1 HSPD1P4 HSPD1P5 HSPD1P6 HSPD1 STAT4 STAT1 HIST2H2BE NEUROD1 CD160 TFRC PHGDH VTCN1 CD2 CD58 SST PTPN22 SSB AP2M1 LAMP3 PSMA5 IFIH1 DPP4 TNFRSF13B VCAM1 IL12A CXCR4 TM4SF1 CCR6 TAGAP IL12RB2 IL23R ESR1 IL1RN IL1B IL1A JUN SUMO4 B3GAT1 SPATA19 IL11 IFNGR1 SRPR IL18RAP SLC4A2 TBPL1 IL1R1 ENPP1 LAIR1 ARG1 CASR CD86 KIR3DL1 AFF3 HSPA8 CD80 CYP2E1 CBL ZAP70 UROD SOCS3 CD200R1 MGAM BTLA MKI67 FYN KLK8 TRAT1 CBLB APOA1 SLC2A1 FOXO3 GRB2 NCAM1 CD8A IL18 FCGRT VAX1 CD300C GNLY IRF5 ATM SLN BAX MMP8 REGL REG1A LILRB1 FLNB FOXP2 TYR TYRL TOR1A PGLYRP1 PPP2R4 F8 SH3GLB2 CTAG1B CTAG1A GAB2 LCK PTPN11 SCD DST XRCC1 GOLGA2 EPB41 LCN2 MPO IL17A CD79A EPO TGFB1 GABRQ DNTT NR0B2 FGF3 VEGFA HSP90B1 CDR1 TCIRG1 RHOA CD40LG MAF CPSF4 FAS TBX21 ACTN4 CDC42P2 CDC42 TLR4 EPCAM OPRL1 KRIT1 CCR5 CCR1 CCR3 CXCR6 PLS3 TXN CPM PADI4 MDM2 DDX41 TSC22D3 IFNG RNF128 TH1L BAD STAT3 PLCB3 STAT5B MEF2A POR BAAT CTNNB1 NODAL CCR8 CCL17 CCL22 LCP2 IQGAP1 LAT2 ANPEP VEGFC BTK CCR7 NFATC2 CEBPB IL12B TNFRSF1B CSF3 TNFRSF8 STAT6 ITK HAVCR2 ERBB2 NOD2 MLC1 CCL25 CXCL10 RCVRN CYP19A1 IFNB1 IFNA1 COPS5 MUC16 CD83 PCSK1 RAB27A RTEL1 HM13 BCL2L1 CDS1 ICAM1 IL7 SMG1 ICAM3 TYK2 HDAC9 APC CA2 VDR TIMP1 SPP1 IL6 BTN3A1 SNCA CD59 ITCH CDR2 IL2RB CD72 GAD2 APBB1IP CD44 IL3 CSF2 EARS2 SLC22A4 SLC22A5 NFKB1 IL5 IL13 IL4 CD82 SYP TNFRSF18 CLTA TNFRSF4 PPARG FOXP3 TSHR TPO HSPA4 IL4R RAF1 LEF1 IL9 NR4A1 CREM CD19 LAT SRC ANXA5 EXT1 TNFRSF14 CD276 IL2 IL21 HLA-C INTU NF1 ATAD2 CD14 ALOX5 MAPK3 SERPINA1 CYP2D6 ITGAL IL15 CCL2 CCL11 TNFRSF25 RBP3 CCL1 TG MIF ADA SLA |

| | |
|---|---|
| | MAPK8 IL2RG MYO9B EOMES TNFRSF9 SDC4 CCL5 CCL15 NR3C2 CCL18 CCL3 CCL4 CD63 MORF4L1 MORF4 ITGAM SILV ITGAX CDK2 JAK3 INSL3 CCR4 GLB1 CXCR3 MS4A2 ACACA IL12RB1 IL16 MS4A1 CD40 CD74 MTOR GUSB IL23A AKT1 GLS2 MTHFR RASGRF2 DHFR DHFRP1 CIITA LIF ACTB ESR2 FCER2 IL8 LYN GATA3 ALB ITGB2 ENAM PFKFB3 VAV1 IL2RA TP53 TNFSF9 MLANA CD68 JAG1 AIRE ICOSLG PDCD1LG2 CTSD CD274 JAK2 PLCB1 FIGF KIT IDO1 CABIN1 FANCB RB1 PRNP ADRA1D TEC TXK AMELX BCR MAPK1 CDKN1B TNFSF11 TRIM21 MBP CXCL16 IL3RA TLR1 KLRK1 KLRC1 KLRD1 EMB NFKBIA CD69 SIRPA EGLN3 CD81 ITGAE PNOC BCL2 INS CD38 RUNX1 CLEC4C ADCY10 GZMB THBS1 HLA-DRB4 IL7R PTPN6 ATN1 TNFRSF10A TNFRSF10B CD4 LAG3 FCGR2B SLC44A4 PSMB9 SMAD7 IRF7 LRPAP1 HBA1 SOD1 HBA2 NEU1 GAPDHL6 GAPDH PRTN3 SETBP1 CD27 TNFRSF1A IRX2 NDN DSG1 TNF RNASE3 DEFB1 ACE THBD TFF3 UCN2 |

**Supplementary Table 3** GO enrichment analysis of integrated DEGs associated with autoimmune thyroid dysfunction and anti-CTLA-4 therapy. DEGs, differentially expressed genes. P value<0.05

a. Hypothyroidism

| Category | Term | ID | Count | P value |
|---|---|---|---|---|
| BP | Negative regulation of apoptotic process | GO:0043066 | 5 | 0.002207313 |
| BP | Negative regulation of mature B cell apoptotic process | GO:0002906 | 2 | 0.004993451 |
| BP | Positive regulation of chemokine (C-X-C motif) ligand 2 production | GO:2000343 | 2 | 0.006238098 |
| BP | Regulation of transcription involved in cell fate commitment | GO:0060850 | 2 | 0.006238098 |
| BP | Mammary gland epithelial cell proliferation | GO:0033598 | 2 | 0.00996315 |
| BP | Regulation of macrophage activation | GO:0043030 | 2 | 0.011201875 |
| BP | Negative regulation of DNA damage response, signal transduction by p53 class mediator | GO:0043518 | 2 | 0.016142034 |
| BP | Negative regulation of intrinsic apoptotic signaling pathway in response to DNA damage by p53 class mediator | GO:1902166 | 2 | 0.016142034 |
| BP | Prostaglandin biosynthetic process | GO:0001516 | 2 | 0.017373395 |
| BP | Positive regulation of B cell proliferation | GO:0030890 | 2 | 0.047684688 |
| CC | Extracellular exosome | GO:0070062 | 12 | 9.01E-05 |
| CC | Perinuclear region of cytoplasm | GO:0048471 | 5 | 0.00503401 |
| CC | Endoplasmic reticulum | GO:0005783 | 5 | 0.013647179 |
| CC | Golgi apparatus | GO:0005794 | 5 | 0.015690594 |
| CC | Extracellular space | GO:0005615 | 6 | 0.016428449 |
| CC | Cell surface | GO:0009986 | 4 | 0.023360004 |
| CC | MHC class II protein complex | GO:0042613 | 2 | 0.025061062 |
| CC | Extracellular region | GO:0005576 | 6 | 0.032887842 |
| CC | Integral component of lumenal side of endoplasmic reticulum membrane | GO:0071556 | 2 | 0.032908793 |
| CC | Extracellular matrix | GO:0031012 | 3 | 0.045072462 |
| MF | Identical protein binding | GO:0042802 | 7 | 1.70E-04 |
| MF | Drug binding | GO:0008144 | 3 | 0.003606113 |
| MF | Protein binding | GO:0005515 | 16 | 0.031494362 |
| MF | Transcription factor binding | GO:0008134 | 3 | 0.043909997 |

b. Hyperthyroidism

| Category | Term | ID | Count | P value |
|---|---|---|---|---|
| BP | Regulation of immune response | GO:0050776 | 4 | 5.92E-04 |

| | | | | |
|---|---|---|---|---|
| BP | Regulation of transcription involved in cell fate commitment | GO:0060850 | 2 | 0.004755668 |
| BP | Macrophage derived foam cell differentiation | GO:0010742 | 2 | 0.005704254 |
| BP | Immune response | GO:0006955 | 4 | 0.006872 |
| BP | Negative regulation of DNA damage response, signal transduction by p53 class mediator | GO:0043518 | 2 | 0.01232066 |
| BP | Negative regulation of intrinsic apoptotic signaling pathway in response to DNA damage by p53 class mediator | GO:1902166 | 2 | 0.01232066 |
| BP | Regulation of glucose metabolic process | GO:0010906 | 2 | 0.020766825 |
| BP | Cell maturation | GO:0048469 | 2 | 0.033770827 |
| CC | Extracellular exosome | GO:0070062 | 10 | 1.98E-04 |
| CC | External side of plasma membrane | GO:0009897 | 4 | 7.88E-04 |
| CC | Macrophage migration inhibitory factor receptor complex | GO:0035692 | 2 | 0.001755204 |
| CC | Extracellular space | GO:0005615 | 6 | 0.004800076 |
| CC | Endoplasmic reticulum | GO:0005783 | 4 | 0.03362098 |
| CC | Golgi apparatus | GO:0005794 | 4 | 0.037363639 |
| MF | Drug binding | GO:0008144 | 3 | 0.002304232 |
| MF | Iron ion binding | GO:0005506 | 3 | 0.009010387 |
| MF | IgG binding | GO:0019864 | 2 | 0.010379714 |
| MF | Identical protein binding | GO:0042802 | 4 | 0.031631477 |
| MF | Cytokine receptor activity | GO:0004896 | 2 | 0.033595541 |
| MF | RNA polymerase II transcription factor activity, ligand-activated sequence-specific DNA binding | GO:0004879 | 2 | 0.033595541 |
| MF | Oxygen binding | GO:0019825 | 2 | 0.043647885 |

**Supplementary Table 4** KEGG pathway analysis of common differentially expressed genes of autoimmune thyroid dysfunction and anti-CTLA-4 therapy

| **Category** | **Pathway** | **ID** | **Gene count** | **P value** | **Genes** |
|---|---|---|---|---|---|
| Hypothyroidism | Tuberculosis | hsa05152 | 3 | 0.048909812 | MAPK1, CEBPB, CD74 |
| | Thyroid cancer | hsa05216 | 2 | 0.057482735 | MAPK1, PPARG |
| | Focal adhesion | hsa04510 | 3 | 0.064120305 | MAPK1, FLNB, SPP1 |
| | Prion diseases | hsa05020 | 2 | 0.0670778 | MAPK1, PRNP |
| Hyperthyroidism | Osteoclast differentiation | hsa04380 | 4 | 0.001309393 | FCGR2B, PPARG, FCGR3A, BTK |
| | Tuberculosis | hsa05152 | 3 | 0.036680203 | FCGR2B, FCGR3A, CD74 |
